# Unpacking Detrimental Effects of Network Externalities on Privacy Invasion, Communication Overload and Mobile App Discontinued Intentions: A Cognition-Affect-Conation Perspective

**DOI:** 10.3390/bs13010047

**Published:** 2023-01-05

**Authors:** Hua Pang, Yang Ruan, Yiwei Wang

**Affiliations:** 1 School of New Media and Communication, Tianjin University, Tianjin 300072, China; 2 College of Management and Economics, Tianjin University, Tianjin 300072, China; 3 Department of Linguistics, University of Constance, 78464 Constance, Germany

**Keywords:** mobile app, network externalities, privacy invasion, detrimental effects, discontinued intentions

## Abstract

Recently, mobile apps are rapidly emerging as an important information instrument, with the potential to boost convenience and efficiency in everyday life. The adoption of mobile apps can exert a positive influence on individuals, but also lead to adverse perceptions in different ways. The crucial issue arising is what motivates people’s discontinued use of such services. Furthermore, the roles of communication overload and privacy invasion between network externalities and discontinued use intentions have not been thoroughly examined. The primary objective of this article is to investigate if negative network externalities may result in privacy invasion, communication overload and discontinued intentions, and how the underlying mechanism operates. This current research collected and evaluated data from 696 mobile app users utilizing the structural equation model (SEM) technique. The findings demonstrate that perceived critical mass and perceived complementarity positively affect the privacy invasion of mobile app users. Particularly, it was discovered that privacy invasion and communication overload mediate the association between network externalities and mobile app discontinued use intentions. This article may not only enrich the ongoing contemporary critical discussion on new information technology usage, but also offer significant theoretical and practical implications for mobile app researchers and practitioners.

## 1. Introduction

In the past few decades, digital transformation has altered the way businesses conduct their operations, develop relationships with customers, suppliers, and other stakeholders, and foster the innovation of business models and the creation of customer value [1,2,3]. The term digital transformation refers to the process by which an organization uses digital technology to construct a new digital business model that assists the organization in creating and appropriating more value for the organization [1,3]. The businesses processes, operational routines, and organizational competencies are all impacted by this transition. The typical relationship that occurs between customers and companies is being reimagined by digitalization [3,4]. Especially, customers may choose from a wide variety of media platforms and interfaces to engage with businesses and other customers actively and easily.

Among the most popular digital marketing channels, mobile applications (apps) such as Facebook, YouTube and WeChat have emerged as critical platforms for people’s everyday communications and offer low-cost ways to reach a large audience and disseminate product and service information online [5,6,7]. A large number of individuals use various mobile apps to constantly engage with others, express feelings, browse daily news, exhibit stunning photographs, or share their living conditions. However, since users are constantly pressured to pay attention to mobile apps and react to an excessive amount of interpersonal requests in a timely manner, physical and psychological strain, known as communication overload, is more likely to appear [8,9]. Previous studies have shown that perceived overload on mobile social media reduces user pleasure and contributes to discontinued intentions [10,11]. Mobile apps are web-based services built on member engagement, and their long-term functioning and growth are mostly on the basis of individuals’ continuance intention [12]. As a result, how to reduce the communication overload may be seen as a strategy for retaining mobile apps users and, therefore, increasing the competitiveness of mobile app providers. Mobile app providers also need to place equal emphasis on figuring out how to keep users and understanding the possible consequences of withdrawal. Among the few scholars who have examined mobile app withdrawal, Lin et al. explored the causes (such as addiction, feelings of guilt, pleasure with sites, and self-efficacy) and effects of discontinuance intention [13]; while Cao et al. analyzed discontinuous use as users’ coping mechanism against stress caused by exhaustion and social overload [14]. Luqman et al. utilized a clinical and vocational perspective and developed the term of social network fatigue to explain discontinuous Facebook usage behavior [15]. They found that the negative emotions of fatigue, boredom, and burnout, generated by mobile app usage, result in discontinuous use behavior among users.

The advantages of using certain products or services are determined by two factors, the total number of existing users and the availability of complementing items [16,17]. There will be a growth in the number of people using the products and the corresponding supplementary commodities, which will lead to the emergence of external advantages. Researchers have frequently mentioned that network externalities may boost the pleasure and usefulness of mobile apps use, which in turn, influences users’ desire to continue using mobile services [6,18,19]. Furthermore, network externalities may have a beneficial impact on users’ identification with social networking sites and their satisfaction with such sites, which can subsequently encourage users’ psychological well-being and commitment [6,20]. These researchers demonstrated that, for individuals, a greater network size in mobile apps and complementary goods, as well as services given by this platform, could result in higher advantages for the users and would attract more participation. In addition, some empirical findings from earlier researchers have shown that network externalities are unable to directly influence the intentions or behaviors of mobile app users [21,22,23]; instead, several mediators, such as pleasure, utility, and contentment are required. Therefore, it is not difficult to demonstrate the fact that these studies mostly focused on the advantageous aspects of network externalities, and that they may have assumed that network externalities would result in favorable outcomes.

Recent research suggests that, for users, the connectivity promoting features of mobile services may also introduce new, unforeseen issues that limit the development of interpersonal networks and have a detrimental influence on the quality of interpersonal relationships [5,6,24]. People who participate in larger social networks are prone to encounter relationships and have more unrestrained connections, both of which are considered to be the main factors that contribute to communication overload [25,26]. With the increasing number of people joined to mobile apps users’ buddy lists, their actual names, universities, interests, and other private information given when they enrolled for personal accounts, would be revealed to others [17,27,28]. As a result, certain privacy security issues may arise. These studies show that the growing user base and complementing technologies could not only produce benefits but also trigger a series of negative outcomes including perceived overload and certain concerns [27,28]. Meanwhile, previous research showed that the antecedents of discontinued intentions caused by a mobile app are directly linked to these effects [24,29]. Consequently, negative network externalities may also be used to probe mobile app discontinued intentions.

Despite how recent research has determined various variables and motivations contributing to discontinuous intentions, including environmental stimuli such as perceived overload [13,24], and psychological factors such as exhaustion and discontent [8,30,31], very few investigations have explored the influence of network externalities of mobile apps, such as perceived critical mass and perceived complementarity, on individuals’ emotions and behavioral reactions. Furthermore, users’ perceived overload have been discovered as a predictor of social network fatigue [7,29]. The majority of past research has claimed that social network fatigue acts as a moderator in the linkage between perceived overload and discontinuous intentions [30,32,33]. However, it is unclear whether privacy invasion and communication overload have a mediating effect on the impact of network externalities on mobile app discontinuous intentions. There have been few researches that utilized the cognition-affect-conation paradigm in the area of cognitive psychology to investigate the possible impact of privacy invasion and communication overload on discontinuous intentions in the setting of mobile apps. Furthermore, although privacy invasion is undoubtedly a serious problem [27,34], very few studies have systematically uncovered the circumstances under which it is most likely to result in discontinuous intentions.

To address this vacuum, the primary objective of this study was to investigate whether negative network externalities, such as perceived critical mass and perceived complementarity, contribute to mobile app users’ discontinued intentions through communication overload and privacy invasion. This research attempts to elucidate the potential influence of cognitive and emotional aspects on mobile app users’ intentions to stop using. From a cognitive psychology perspective, the study offers pertinent hypotheses and a conceptual model based on the cognition-affect-conation framework and cognitive load theory. In addition, the mediating impacts of communication overload and privacy invasion are also investigated. This current article makes some significant contributions to the literature on network externalities and discontinuous intention by investigating mobile app users’ privacy invasion and communication overload and the factors leading to these. Specifically, first, while previous studies have documented various factors influencing mobile app discontinuous usage intentions [15,26,29,31], the influence of network externalities has never been investigated. This empirical research identified perceived critical mass and perceived complementarity as the new antecedents of discontinuous intention of mobile app usage. Second, although perceived overload has been discovered to potentially trigger mobile app fatigue and information avoidance behavior [10,24,30], the article validates and extends previous research by demonstrating that communication overload and privacy invasion can also result in users’ discontinuous intentions. In addition, this study extends previous research by verifying that communication overload and privacy invasion mediate the impact of perceived critical mass and perceived complementarity on discontinuous intention. Third, by examining the moderating effect of communication overload and privacy invasion, this work provides a more comprehensive understanding of the mechanisms underlying mobile app discontinuous use intentions. These findings will assist mobile app providers in better understanding users’ emotions, usage behaviors, and the variables affecting their use of mobile apps. This knowledge may also help mobile app providers to sustain the active use of mobile apps in the contemporary digital media era.

## 2. Theoretical Framework and Hypotheses Development

### 2.1. Linking Network Externalities to Privacy Invasion

In order to accommodate as many users as possible on the available resources, mobile app providers always strive to maximize their web-based social networks, which will eventually lead to the emergence of network externalities [18,19,35]. Network externalities are defined as the value or impact that users gain from a product or service that will bring about greater value to consumers with the rise of users, complementary goods, or services [17,36,37]. Externalities in networks are concerned with the elements that produce network effects, such as network size and complementary commodities or services [19,38]. According to this conventional definition, while investigating network externalities under the environment of mobile services, researchers mostly concentrated on their positive aspects and uncovered various advantages brought on by the larger network sizes on mobile apps [16,21,22]. However, the user’s own utility is always subject to the effect of the techniques used by other users. Users will constantly want to maximize their own utility because of the individual rationality and self-centered character of humans. Within the context of resource sharing, it is unavoidable that the optimum strategies of other users will be impacted by negative network externalities. In a nutshell, the impact of negative network externalities will take place when there are more users of a resource, which will result in the resource’s decreased value.

In general, there are two categories of network externalities: direct and indirect dimensions [17,37]. Direct externalities for mobile app users are associated with the total number of people using the network, whereas indirect network externalities place greater emphasis on the benefits gained through complementary products and services [19,39]. Thus, in the setting of the mobile app, perceived critical mass and perceived complementarity were utilized in some prior studies to describe the different aspects of network externalities, respectively [37,39,40]. The first relates to the total number of mobile app users, and the second, to various auxiliary features or supporting tools that are integrated into the mobile app, such as voice messaging, picture sharing, location-based services, and even online commerce [35,39]. Although the consequences of negative network externalities are the major focus of this study, their magnitude is still limited by the number of current users and ancillary commodities [21,38]. The primary difference between this study and other studies is that, instead of concentrating on advantages, this study largely concentrates on certain adverse effects brought on by the rise in users or supplementary technologies. Perceived network size and perceived complementarity were still utilized to depict the consequences of negative network externalities in the setting of mobile apps.

With a growing number of users added to a mobile app user’s buddy list, the user’s personal information when he or she registered for their personal accounts, or that they freely uploaded, is more likely to be seen or disclosed to other persons and third parties [17,22,26]. It is not difficult to comprehend that the mobile app is a linked world, and that the number of potential sources of personal information breaches is substantially expanded inside it, as the people connected to an individual’s network are nodes within a wider network [16,38]. Over the past ten years, the ubiquity of Internet and mobile technologies has made information privacy a pressing concern for new technologies such as cloud services, mobile applications, location-based services, and electronic commerce [17]. Furthermore, additional mobile app integrated supplementary products or services, such as albums, likes, and picture exchanging, might result in privacy disclosure [28,41]. These technologies can not only enhance users’ social connections, but also assist them in more successfully seeking and obtaining various types of information. More significantly, as mobile devices become more prevalent, further privacy problems may arise when new technologies are included in these platforms [12]. If mobile app members publish or exchange content, the global position system capabilities of smartphones and tablets might reveal their whereabouts and other demographic data. As a result, the following two hypotheses are advanced:

**H1.**  *Perceived critical mass is positively associated with privacy invasion*.

**H2.**  *Perceived complementarity is positively associated with privacy invasion*.

### 2.2. Linking Network Externalities to Communication Overload

Typically, communication overload is defined as a circumstance in which the communication needs of a network outnumber an individual’s communication ability, which might disrupt a user’s study or work schedule [30,42]. Communication overload can disrupt users’ typical routines, making it difficult for them to concentrate [30]. This might lead to a decrease in judgment accuracy, which can have a detrimental impact on users’ feelings about trusting the information and even other people. Simultaneously, when confronted with social interactions that must be dealt with, users who lack adequate communication skills may be at a loss and may experience exhaustion and anxiety [17,29]. Some scholars have suggested that humans are cognitively able to regulate a finite number of connections concurrently, and if that number is surpassed, they are no longer able to manage them, resulting in communication overload [28,42]. The mobile app may be considered a virtual platform, and communication overload is unavoidable. As the use of the mobile app continues to increase, more members experience the adverse impacts of population density, such as excessive self-presentation and socializing, as well as connections.

On mobile apps, multiple channels of electronic communication with web-based friends and followers create a large amount of shared information. People are more likely to have a wide variety of near and distant connections, as opposed to the more limited number in offline social networks [14,43]. This implies that users may be subjected to unwelcome human networks or an excessive volume of social messages, and if they are unable to successfully cope with the situation, they may perceive overload from using these sites. Several factors may account for this: initially, it will be challenging to cope with certain undesirable human networks [9,42]. Users will progressively experience mental weariness as they adopt various techniques to deal with unpleasant social encounters. In addition, communication overload could be deemed as the perception or belief among mobile app users that they must expend an inordinate amount of time and effort to react to social demands and sustain interpersonal interactions in developing web-based networks [5,13].

Although intense mobile app use is linked to greater perceptions of emotional support that bring users closer to their friends, users may encounter more social requests that call for specific types of reactions when more friends join the same mobile app [8,24,31]. That implies they will need to devote more time and attention to the management of these social ties, which may ultimately result in communication overload. Frequent communication in a continually connected world with an expanding social network may result in an imbalance between communication needs and human cognitive capacity [9,14]. Users become overwhelmed by communication overload because they are unable to handle the situation effectively. Simultaneously, numerous new functionalities have been inserted in mobile apps with the help of supporting technologies offered by smartphones, making interpersonal interactions more effective and entertaining [8,15]. These new supporting platforms could make it easier for users to communicate more effectively with their online friends; however, this would also lead to an increase in the number of additional social interactions and demands for social support, both of which need to be dealt with and managed in a timely manner. In light of this, the following two hypotheses have been put forward:

**H3.**  *Perceived critical mass is positively associated with communication overload*.

**H4.**  *Perceived complementarity is positively associated with communication overload*.

### 2.3. Linking Privacy Invasion and Communication Overload to Discontinued Intentions

Prior research has verified that the three sequential phases in a mobile app’s life cycle are adoption, use, and termination [5,17]. Discontinuance is a postcontinuance choice to quit using a continuing platform [33,44]. Continuing to utilize the mobile app will only affect the use phase of its life cycle, while stopping will affect the end stage. As a result, if merely continuation is considered, we can only partly understand what occurs in the latter phases of the postadoption process. Recent empirical studies have established that diverse sets of causal processes are responsible for mobile app continuation and cessation [31,42]. It is clear that discontinuance is not just the opposite of continuity. This notion of mobile app discontinuation intentions refers to users’ future plans to quit using a certain mobile app service temporarily or permanently [29]. From the holistic, theoretical perspective, including social cognitive theory and the stressors–strain–outcome paradigm, most of the previous studies have typically deemed discontinuance as users’ adaptation behaviors in response to negative perceptions resulting from their usage, and have explained how discontinuance intention is shaped [11,26,42].

Numerous mobile app users have reported concerns that their privacy would be compromised by the platform; however, these worries have not impeded media usage [16,24,29]. The privacy paradox is the conflicting link between the attitude of privacy and the conduct of usage. When users understand that their privacy is watched, leaked, or otherwise violated, they may diminish or even cease utilizing related media. Communication overload has been connected favorably with privacy concerns, self-disclosure, parental encouragement, and anxiety [8,26]. Communication overload is the propensity for individuals to disengage from social media activity, which correlates substantially with privacy concerns [24,42]. However, the link between privacy issues and discontinued intentions has not been established. According to the cognitive-affective-conation framework [5], behavioral intention is one of the components of attitude, although privacy invasion as an emotional factor may result in passive use intention. The more privacy issues there are, the more probable it is that users will have passive use intentions.

Some scholars have shown that users have substantial incentives and flexibility to halt social media usage when confronted with stressful conditions [19,24,44]. Informational irrelevance, information overload, and social overload have been shown to have favorable impacts on information avoidance behavior [5,10]. Technostress was only one example of a technological element or function that was available to mobile app users. The aforementioned research shows that user behavior might change depending on the stressor [33,41,44]. As a result, we speculate that users’ plans to discontinue the use of a mobile app are really a set of coping mechanisms designed to reduce the impact of that pressure. A user’s goal for discontinued use may be indicative of a desire to limit involvement with the platform due to a sense of communication overload [31,44]. Accordingly, the following hypotheses are put forward:

**H5.**  *Privacy invasion is positively associated with mobile app discontinued intentions*.

**H6.**  *Communication overload is positively associated with mobile app discontinued intentions*.

### 2.4. Linking Network Externalities to Discontinued Intentions

Perceived critical mass represents the number of a user’s friends and peers who have adopted a mobile app [16,17]. When the size of the network is vast, a user is able to communicate with more peers and acquaintances. This may enhance a user’s perception of the utility of the mobile app. Perceived critical mass may also have a direct effect on discontinued use [13,44]. Excessive social engagements on a mobile app drive individuals to continually respond to their online friends, requiring them to devote tremendous effort [15]. Excessive social demands may also disrupt and divert users’ attention away from their everyday activities, resulting in avoidance behavior [10,33]. In addition, when users receive a notification, they may consider how much time and effort they must invest in it, leaving them feeling exhausted [30]. Users may even begin to fear the notifications they receive on a regular basis, which can cause stress or other unpleasant feelings. In addition, many users sustain a sizable number of social connections that surpass their cognitive limits [14,26]. When the sustained social relationship exceeds a certain level, the extra-social relationship causes a deterioration in psychological health. Psychological research has demonstrated that psychological exhaustion can reduce engagement and performance, increase the propensity for behavior change, and have negative effects on individuals’ continuous behaviors [5,32,45].

Perceived complementarity indicates how a consumer perceives the availability of complementary goods or services [16,38]. As the number of users grows, service providers may add more features and apps to their mobile app. These value-added features and services may increase the perceived utility of the mobile social app by providing users with access to a multitude of functions on a single platform [40]. However, perceived complementarity may directly influence users’ inclination to discontinue use. When a user obtains extensive functions and services via a mobile SNS, rapidly updated system features have become one of the external stressors for them [13,44]. For instance, some mobile apps upgrade their user interface and system functions virtually weekly [40]. New mobile app features need considerable amounts of time to master before they can be utilized, resulting in varying levels of exhaustion among users [24]. Meanwhile, users may have been acclimated to a certain appearance and find changes to the system to be overwhelming. Even if the upgraded functions and features satisfy user requirements, these functions and features must be simpler to use than their predecessors. Otherwise, users may experience technological overload and become exhausted by the mobile app [8,9]. Unwanted user interface updates will increase people’s sense of fatigue with the platform. Moreover, emotions of tension and tiredness, which are closely associated with social exhaustion, will also contribute to discontinuance [14,15]. Multiple studies have suggested that network externalities are one of the most influential environmental variables influencing discontinuance of new technology [5,11,24]. Fu et al. demonstrated that mobile app users were exposed to a variety of technological characteristics or functions, such as technostress, which influenced their involvement behavior [42]. Similarity, Li et al. found that users had substantial incentives and freedom to halt mobile app use in stressful situations [13]. Following the above discussion, the following hypotheses are proposed:

**H7.**  *Perceived critical mass is positively associated with discontinued intentions*.

**H8.**  *Perceived complementarity is positively associated with discontinued intentions*.

## 3. Research Methodology

### 3.1. Research Model

**Figure 1:**
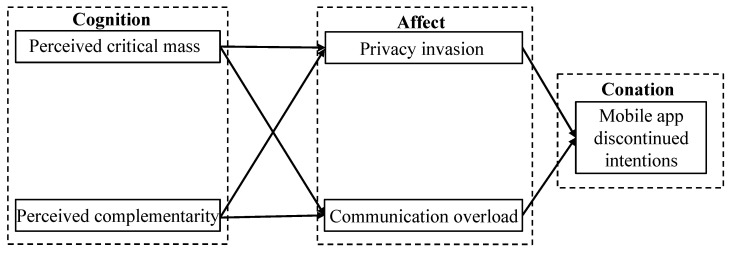
The proposed conceptual research model.

Cognitive load theory asserts that the effectiveness of learning and problem-solving declines when an individual requires additional cognitive resources to understand knowledge than they possess [46,47]. Moreover, overload is the primary element that hinders the utilization of information and communication technologies [11,24]. Overload may absorb users’ cognitive resources, resulting in negative emotions and inactive use. According to the cognition-affect-conation (CAC) framework, cognition is the process by which an individual forms a belief about the outside world; affect is the subjective feeling that an individual experiences while interacting with something or someone; and conation is the ultimate behavioral intention based on that individuals’ perception and psychological feeling [43,48]. In this investigation, users’ subjective cognition of the surrounding environment will have an effect on individual mood, which in turn will create conation. The article hypothesizes that users’ perceived critical mass and perceived complementarity impact communication overload and privacy invasion, which ultimately contributes to mobile app discontinued intentions. Figure 1 depicts the conceptual model according to the preceding study hypotheses.

### 3.2. Sample and Data Collection

The initial steps included carrying out a pilot study, in which 50 students were asked to take part in the survey. The study participants were people who utilized a mobile app, and their responses to the questionnaire contained input that was included into the development of the assessment questions. For data collection, the final version of the Chinese questionnaire was posted on a popular online questionnaire website https://www.wjx.cn/ (accessed on 8 August 2022) and links to the questionnaire were sent to popular mobile apps such as WeChat and Weibo. The redesigned questionnaire was circulated during a two-month period in 2022. All respondents were informed in advance about the general purpose of the study and assured that their responses would be voluntary and anonymous. The study protocol was approved by the ethics committee of Tianjin University. Before the main survey, the study carried out a modest pre-survey to confirm that the questionnaire was scientific and effective. The duplicate samples and randomly completed questionnaires were checked out and discarded once the surveys were collected. Furthermore, surveys that lasted less than one minute were deleted, yielding a total of 696 valid questionnaires. Table 1 displays the findings of the descriptive statistical analysis. The majority of responders were between the ages of 21 and 39. Men made up 54.0 percent of the sample, while women accounted for 46.0 percent. Approximately 24.1 percent of the respondents had been using mobile apps for 3–4 years, and nearly 52.0 percent had been using these platforms for more than 5 years. Most of the respondents spent more than three hours daily on mobile apps friends (63.6 percent), whereas only 4.6 percent spent less than one hour per day on these services.

**Table 1:** Summary of participants’ demographics (n = 696).

Measures	Categories	Frequency	Percentage (%)
Gender			
	Male	376	54.0
	Female	320	46.0
Age			
	Under 20 years old	25	3.6
	21–29 years old	388	55.7
	30–39 years old	263	37.8
	More than 39 years old	20	2.9
Educational level			
	Middle school or below	32	4.6
	High school	76	10.9
	Undergraduate degree	369	53.0
	Postgraduate degree	212	30.4
	Doctoral degree	7	1.1
Monthly expenditures			
	Under 2000 RMB	238	34.2
	2000–6000 RMB	192	27.6
	6001–8000 RMB	136	19.5
	8001–11,000 RMB	98	14.1
	More than 11,001 RMB	32	4.6
Using experience			
	Less than 1 year	10	1.5
	1–2 years	30	4.3
	2–3 years	126	18.1
	3–4 years	168	24.1
	More than 5 years	362	52.0.
Daily hours spent			
	Less than 1 h	32	4.6
	1–2 h	96	13.8
	2–3 h	125	17.9
	3–4 h	187	26.9
	More than 4 h	256	36.8

**Table 2:** Fit indices for the measurement model.

Model Fit Measures	Model Fit Criterion	Index Value	Good Model Fit (Y/N)
Absolute fit indices			
RMSEA	<0.08	0.062	Y
RMR	<0.05	0.028	Y
χ^2^/d.f. (χ^2^ = 566.261, d.f. = 238)	<3	2.379	Y
Incremental fit indices			
CFI	>0.9	0.955	Y
AGFI	>0.8	0.809	Y
IFI	>0.9	0.986	Y
TLI	>0.9	0.926	Y

### 3.3. Measurement

The questionnaire is divided into two sections: the first section contains the question items for all of the components in the conceptual model; the second section inquires about the demographic characteristics of the respondents, including their gender, age, level of education, monthly expenditure, using experience, and daily hours spent. The measures utilized to evaluate these constructs were taken from previous investigations and with some slight adjustments to improve the validity and fit into the study environment.

#### 3.3.1. Perceived Critical Mass

The three-item measure was restructured from extant studies to reflect participants’ perceived critical mass [16,37]. Sample items include: “The mobile app seems to be widely used among the people I know”, “I believe that the majority of my friends have a mobile app account”, and “I believe that, in the future, many friends will use the mobile app”. All of these questions were created utilizing a 5-point Likert scale (1 = strongly disagree, 5 = strongly agree). Cronbach’s alpha was 0.858, indicating excellent reliability (M = 3.54, SD = 1.06).

#### 3.3.2. Perceived Complementarity

This study employed four questions from the previous study concerning perceived complementarity [19,37]. The specific questions include the following statements: “The mobile app offers a diverse set of services”, “The mobile app provides a broad variety of helpful features”, “On the mobile app, you may participate in a variety of social activities”, and “The mobile app offers a variety of friend-finding features.” To measure these items, the study used a 5-point Likert scale varying from “1 = strongly disagree” to “5 = strongly agree”. The scale suggested a high degree of reliability (Cronbach’s α = 0.92, M = 3.84, SD = 1.15).

#### 3.3.3. Privacy Invasion

Respondents’ privacy invasion was gauged on three questions revised from prior scales [5,49]. Statements were composed of “Personal accounts are readily leaked due to the features of the mobile app”, “The usage of the mobile app increases the possibility of personal data leaking”, and “Personal data authorization bothers me while utilizing the mobile app”. All items were gauged based on a 5-point Likert scale from 1 to 5, representing strongly disagree to strongly agree. Cronbach’s alpha was 0.825, which exhibited good reliability (M = 3.66, SD = 1.13).

#### 3.3.4. Communication Overload

The participants reported on a group of statements related to communication overload [8,9]. Certain questions included the following statements: “I get too many messages from friends or acquaintances on the mobile app”, “I have to send more mobile app messages than I want to”, “I receive too many mobile app alerts while doing other chores”, “Mobile app messages overwhelm me” and “I can’t keep up with friends’ mobile app messages and news”. All questions were graded on a 1-to-5 Likert scale, from strongly disagree to strongly agree. Cronbach’s alpha was 0.930, indicating high reliability (M = 3.85, SD = 1.16)

#### 3.3.5. Mobile App Discontinued Intentions

Three questions from a relevant study were carefully edited and used to assess the intention to discontinue app usage [29,32]. Participants were asked to what extent they agree these three items: “I occasionally leave the mobile app and return later”, “I expect to cease using the mobile app within the next three months” and “I intend to discontinue using the mobile app within the next three months”. These three items were examined according to a 5-point Likert scale ranging from “1 = strongly disagree” to “5 = strongly agree” (Cronbach’s α = 0.875, M = 3.55, SD = 1.18).

## 4. Data Analysis Strategy

The current article adopted structural equation modeling employing the AMOS 23.0 program to assess the measurement and structural model. AMOS is a sophisticated approach that combines principle components analysis (CFA) with regression to estimate both this measurement and the structural model at the same time [50,51]. AMOS is also more capable of dealing with formative measurements and moderating relationships [20,52]. According to some scholars, AMOS is not only capable of formulating a formative model for latent constructs, but also has a few prerequisites for verifying a model and displaying its graphical interpretation [10,51]. Consequently, the study used AMOS, Version 23.0, software for Computer Program; Chicago, IL, USA: IBM SPSS, 2014. to analyze both the CFA and structural models in this investigation.

## 5. Results

### 5.1. Measurement Model

In the beginning, confirmatory factor analysis (CFA) was used to test the suggested model. AMOS 23.0 was utilized to examine the measurement model made up of overall model fit, construct reliability, and validity. After that, structural equation modeling, often known as SEM, was carried out to examine the connections in an intuitive manner. The researchers chose a two-step technique to assess the suggested model and the link between the study’s variables since it ensures measurement reliability and validity and makes the findings more significant than utilizing just one approach [5,26]. The first stage was to analyze the measurement model, and the second step was to explore the structural links between all components.

The model was assessed by absolute fit indices (χ^2^/d.f. = 2.379; RMSEA = 0.062; RMR = 0.028) and the incremental fit indices (CFI = 0.955; AGFI = 0.809; IFI = 0.986; TLI = 0.926). Table 2 displays the findings, which imply an adequate model fit. Cronbach’s alpha and composite reliability (CR) are markers used to demonstrate a structure’s internal consistency. All of the Cronbach’s alpha and CR values were higher than the acceptable value of 0.7, which shows that the data was reliable. Similarly, an analysis of factor loadings, the average variance extracted (AVE), and squared multiple correlations (SMC) were used to confirm convergent validity. The strong loading on the component confirms the excellent convergent validity of the potential construct. The loading values, ranging from 0.766 to 0.889, were more than 0.7, indicating high convergent validity. Every construct’s AVE exceeded 0.5, indicating acceptable convergence. The suggested measurement model’s convergent validity was shown by SMC values greater than 0.5. Table 3 presents some statistical findings on the confirmatory factor analysis. As indicated by some scholars [17], the estimated values of AVE should be compared to the squared correlations of other constructs. Every AVE (diagonal terms) in Table 4 is greater than the associated squared correlation coefficients (off-diagonal terms), indicating excellent discriminant validity. As a result, this measurement model demonstrates adequate model data fitting, acceptable reliability, and plentiful convergent and discriminate validity.

### 5.2. Structural Model

**Figure 2:**
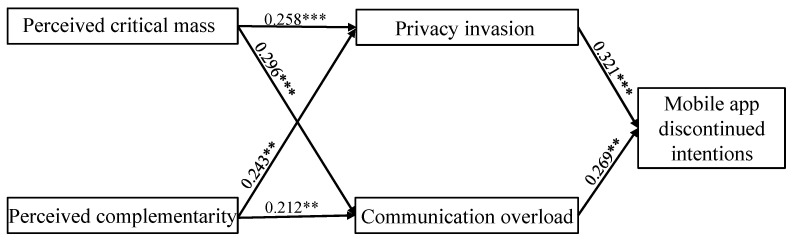
Path analysis result of the structural model. Note: ** *p* < 0.01; *** *p* < 0.001.

The structural model was verified utilizing data from the validated measurements. The suggested model’s overall fit indices are acceptable since the findings fall within commonly recognized ranges. The chi-square/df is 2.122, RMSEA is 0.052, NFI is 0.959, IFI is 0.989, TLI is 0.938, and CFI is 0.969. Given that the data revealed an adequate model fit, we computed the route coefficient. The outcomes demonstrated that the computed path coefficients were significant. The findings showed that perceived critical mass (b = 0.258, *p* < 0.001) and perceived complementarity (b = 0.243, *p* < 0.01) have a significant positive association with privacy invasion. Thereby, H1 and H2 are accepted. Perceived critical mass (b = 0.296, *p* < 0.001) and perceived complementarity (b = 0.212, *p* < 0.01) exert a significant positive association with communication overload. Thus, H3 and H4 are supported. Moreover, privacy invasion has a significant positive association with mobile app discontinued intentions (b = 0.321, *p* < 0.001), thereby supporting H5. The association between communication overload and mobile app discontinued intentions is significant (b = 0.269, *p* < 0.01), thereby supporting H6. Finally, this article explores the mediating effects of privacy invasion and communication overload between perceived critical mass, perceived complementarity and mobile app discontinued intentions. The bootstrapping findings verify that the linkage between perceived critical mass and mobile app discontinued intentions is partially mediated by privacy invasion (b = 0.081, *p* = 0.012 < 0.05; 95% confidence interval (CI) = [0.010, 0.190]) and communication overload (b = 0.067, *p* = 0.036 < 0.05; 95% confidence interval (CI) = [0.030, 0.180]). Similarly, the bootstrapping results also demonstrate that privacy invasion (b = 0.038, *p* = 0.015 < 0.05; 95% confidence interval (CI) = [0.030, 0.170]) and communication overload (b = 0.042, *p* = 0.029 < 0.05; 95% confidence interval (CI) = [0.022, 0.130]) could partially mediate the association between perceived complementarity and mobile app discontinued intentions. Hence, H7 and H8 are supported. As a result, the study concludes that the proposed model is acceptable (Figure 2).

## 6. Discussion

### 6.1. Summary of Key Findings

Building upon the cognition-affect-conation (CAC) framework [46,48], this research investigates how perceived critical mass and perceived complementarity lead to privacy invasion and communication overload, and how these factors affect mobile app discontinued intentions. The study examines the direct and indirect potential consequences of perceived critical mass and perceived complementarity on mobile app discontinued intentions, with privacy invasion and communication overload acting as moderators. The obtained results support the six hypotheses and lead to some intriguing discoveries.

Firstly, the results discover that perceived critical mass and perceived complementarity are both positively correlated with privacy invasion. This positive association is consistent with the evidence from existing studies, which confirmed that the discontinued intentions to use mobile apps can be predicted [27,40,45]. More specifically, users are prone to evoke privacy issues when they perceive more critical mass and complementarity in the settings of mobile app services. The results corroborate the conclusions of other scholars, who found that the benefit of interactive communication technologies was positively correlated with the total number of people using them, and the provision of complementary services resulted in an indirect externality known as perceived complementarity [17,38]. This indicates that privacy invasion on mobile devices is not only derived from the demand side, but also from the supply side, via the provision of complementary goods and services. In addition, the amount of content, including status updates, images, and other online items, posted by mobile app users is increasing every year. Evidence for the rise of privacy disclosure, which has not been extensively studied in the literature, is provided by the discovery that direct and indirect network externalities may promote the sense of privacy invasion.

Secondly, perceived critical mass and perceived complementarity are found to exhibit significant positive effects on communication overload. This conclusion is consistent with recent study findings. Previous studies on the influences of using mobile devices consistently indicated that the perceived critical mass and perceived complementarity have become the significant factors influencing communication overload [8,34,53]. Additionally, the vast majority of the currently available research concentrates on the causal link between the antecedent factors and the outcomes of privacy invasion and communication overload [19,37], while disregarding the causal relationship between the antecedent variables and mobile app discontinued intentions. Nonetheless, the results of this investigation provide supporting evidence. Consistent with the findings of prior investigations [5,24], communication overload and privacy invasion have a positive effect on discontinued intentions in the computer-mediated environment.

Thirdly, this research adds to the literature by identifying the roles that communication overload and privacy invasion play as mediators between perceived critical mass, perceived complementarity and mobile app discontinued intentions. As the number of users of a mobile app and its related tools continues to rise, these factors will have an effect, both directly and indirectly, on discontinued intentions through concerns around privacy and communication overload. This demonstrates that, from the perspective of the parallel side, cognitive overload is an essential criterion that users adopt when evaluating the system and ultimately determining whether they feel content with it at the stage of post-adoption of information technology [24,28,31]. As a result of this, the findings have shown that persons who experience overload and privacy invasion from mobile apps are more likely to trigger negative responses that result in their ceasing all usage of the technology. Furthermore, our conclusion on discontinuance intention experimentally verifies the finding of A. R. Lee et al., who conducted interviews with social networking service users and discovered that users prefer to change their activities to prevent social network overload [8].

### 6.2. Theoretical and Managerial Implications

The following theoretical implications are provided by this work for future research. First, the results of this research add to the mobile app literature by providing a well-supported conceptual model for communication overload. Although other studies have attempted to examine how perceived overload develops, the focus of this study was on the negative outcomes created by network externalities that might result in discontinuance intentions. Some negative effects, such as privacy invasion, and communication overload, would arise for mobile apps as the number of users and associated supplementary commodities rises. Second, this research broadens the scope of network externalities research in the setting of a mobile app. In contrast to earlier research, this research concentrates on the detrimental aspects of network externalities. Although several scholars have discovered the crucial functions of network externalities on mobile social media, the majority of them have focused on the positive aspects [17,25]. The findings of this study confirm the existence of negative network externalities for mobile app users. As the size of the users and the related tools continue to expand, perceived critical mass and perceived complementarity will exert a direct and indirect impact on mobile app discontinued intentions. It is a truth that additional users reduce the value of accessible resources, which diminishes the utility of users taking the same option. Third, the cognition–affective–conation framework may be applied to the realm of mobile app user behavior. Conceptual models from the area of cognitive psychology may aid researchers in gaining a deeper understanding of discontinued use intentions. Additionally, the model assists researchers in comprehending the emotions and attitudes of mobile app users throughout their response.

The research also yielded potentially significant findings for the users and providers of mobile app services. First, since privacy and communication overload concerns might have an effect on mobile app discontinued intentions, it is vital for mobile app providers to allow users complete control over the contents they publish on these sites, as some of these contents can merely be shared with intimate friends. Moreover, some researchers emphasized that such regulatory mechanisms should not be too complicated due to a number of adverse impacts [40,51,52]. Thereby, making posts public for a limited time would minimize members’ privacy and communication overload concerns by preventing private, informal, or indelicate information from being viewed or gathered by others. Second, in order to assist users in handling their online communication requests and network connections, it is important to include certain features and capabilities directly into platforms. Based on our findings, filter mechanisms may be a viable option for preventing communication overload, which is another key factor in triggering abandonment plans. As a result, mobile app platforms should make it possible for its users to adopt efficient filtering mechanisms in order to cut down on duplicate communication requests. Users who are constantly reported for improper activity, such as harassment or spamming, will have a poor rating and be unable to add contacts. Third, mobile app users may recognize the reasons and forms of abandonment intention to balance social reality and online social networking for a better user experience. When confronted with the various features provided in mobile apps and social relationships, users may utilize capabilities such as contact categorization, group management, and blocking commercial sources, to prevent apparent overload. Mobile app providers may entice and retain users by regularly updating and redesigning their applications, communicating with users about the latest updates, and providing rewards for frequent app use via mobile app marketing. Furthermore, the adoption of different forms of technology, particularly social networks, enables digital startups to create a direct and emotional contact with clients, allowing a firm to gain knowledge of consumer demands, sentiments, and preferences. Digital startups may develop sensing capacities in close interaction with consumers by focusing on the emotive and social components. According to some scholars [1,4], with digital technologies, firms initiate a virtual cycle that begins from customer contact, continues with customer happiness and commitment, and arrives at customer engagement. Digital startups could progressively develop sensing capabilities throughout this process, which improves all aspects of the client’s experience. Moreover, it is feasible for digital businesses to provide a personalized customer experience based on changing consumer demand and by introducing unique promotions [2,3]. This produces a personalized customer experience for the most advanced stage of the value-creation process: customer involvement across the whole customer journey.

## 7. Limitations and Suggestions for Future Research

For a number of reasons, the outcomes of the present research should be cautiously interpreted. First, this research used mobile app users who responded to an online survey as participants. As a result, these samples might not accurately reflect the characteristics of the overall population and might be biased by self-selection. Second, statistical analysis gives numerical connections, and the interpretation of the data is reliant on subjective evaluation; even though the results are in line with accepted hypotheses and conclusions from previous research, which gives the results more credibility. Focus groups have been shown in evaluation research to be an effective way of gathering a wide range of information [54]. Focus groups provide an excellent opportunity to gain valuable insight into the experiences, observations, and opinions of group members [55]. Thus, future studies should also employ the use of techniques such as focus groups to provide revealing outcomes. Third, in addition to overload and privacy issues, additional environmental, self-related, and psychosocial elements also have an impact on mobile app users’ discontinued intentions. Researchers may explore the causes of discontinued use intentions in the future. Additionally, the psychological mechanisms by which negative network externalities influence intentions for discontinued use are not well-understood. Further research may be performed on additional mediating and moderating factors.

## Figures and Tables

**Table 3:** Statistical outcomes of confirmatory factor analysis.

Constructs and Items	Loading (>0.7)	SMC (>0.5)	CR (>0.7)	AVE (>0.5)
Perceived critical mass (PM)			0.847	0.649
PM1	0.766	0.587		
PM2	0.782	0.612		
PM3	0.866	0.749		
Perceived complementarity (PC)			0.913	0.724
PC1	0.852	0.726		
PC2	0.846	0.716		
PC3	0.878	0.771		
PC4	0.826	0.682		
Privacy invasion (PI)			0.830	0.619
PI1	0.801	0.642		
PI2	0.768	0.589		
PI3	0.792	0.627		
Communication overload (CO)			0.923	0.707
CO1	0.778	0.605		
CO2	0.886	0.785		
CO3	0.788	0.621		
CO4	0.856	0.733		
CO5	0.889	0.790		
Mobile app discontinued intentions (MI)			0.868	0.669
MI1	0.771	0.594		
MI2	0.881	0.609		
MI3	0.799	0.587		

Notes: SMC, squared multiple correlations; CR, construct reliability; AVE, average variance extracted.

**Table 4:** Discriminant validity.

	PM	PC	PI	CO	MI
PM	**0.806**				
PC	0.516 **	**0.851**			
PI	0.690 **	0.401 **	**0.787**		
CO	0.536 **	0.421 **	0.677 **	**0.841**	
MI	0.519 **	0.441 **	0.688 **	0.643 **	**0.818**

Notes: ** *p* < 0.001. Diagonal elements (bold) represent the square root of each construct’s AVE. Off-diagonal elements represent squared correlations between variables.

## Data Availability

The data of this study are available from the corresponding author upon reasonable request.
